# Supplementary material for: Mitonuclear interactions shape both direct and parental effects of diet on fitness and involve a SNP in mitoribosomal 16s rRNA
Source: PLoS Biol. 2023 Aug 21;21(8):e3002218. doi: 10.1371/journal.pbio.3002218 (PMC10441796; doi:10.1371/journal.pbio.3002218)
Supplement: S1 Text — Novel high-lipid diet represses fecundity. Text B in S1 Text. Initial fecundity experiments: Specific nutrients sufficient for DMN variation. Text C in S1 Text. Multitrait phenotyping with chronic and parental diet manipulation: Analysis by geographic origin. Text D S1 Text. AIC and r2 calculations. (DOCX) [file pbio.3002218.s001.docx]

**Supplementary text**

1. **Novel high-lipid diet represses fecundity**

Previous studies showed mitonuclear modulation of effects of diluting dietary yeast [1–5], which is a source of multiple nutrients. We studied effects of specific nutrients, normally derived from yeast, by specifically enriching either lipids or essential amino acids (EAAs). EAA enrichment increases fly fecundity ~20% [6–8]. We were interested in lipids because of relevance to Western human diets [9,10], and so we developed a new high-lipid diet (see materials & methods). We characterized impact of this diet on the ancestral population of Beninese wild-type flies from which our experimental populations were derived. These flies were fed on high-lipid food for one week, before laying eggs overnight on development medium, to ensure that any effect of the manipulation reflected physiological capacity to lay eggs, and not oviposition preference. This high-lipid feeding decreased fecundity by 20% (Figure S2A), confirming that this diet modulates reproduction, but inversely mirroring EAA's effect.

1. **Initial fecundity experiments: Specific nutrients sufficient for DMN variation**

The lipid-enriched and EAA-enriched diets were fed to flies as shown in Figure S2B. After >158 introgressions of the flies, EAA and lipid were each sufficient to cause DMN variation in a reproductive metric, fecundity (Figure S2C). Impacts of nutrition are determined both by qualitative variation in diet composition, and quantitative variation in calories [11]. However, among our populations, variation in fecundity could not be accounted for linearly by caloric density, suggesting that EAAs and lipids are specific nutrients that are sufficient to elicit DMN variation (Figure S2D). Diet was therefore modelled as an unordered factor. A DMN interaction was visually apparent (Figure S2C). A statistical model (GLMM, Table S4), which explained the majority of variance (conditional R^2^=0.768), confirmed the DMN interaction (p=0.001). Fecundity variation was technically repeatable among replicate experiments (Figure S2E), without differences that would indicate intergenerational drift.

To assess repeatability among genetic replicates, and to identify differential effects of EAA versus lipid enrichment, we calculated estimated marginal means (EMMs) [12] for each line on each diet. EMMs were visually correlated among genetic replicates (Figure S2C). To assess repeatability, we calculated an index of differences between conditions and p-value for each pairwise diet:line comparison (Figure S2F). First, we used these values to assess consistency among the genetic replicates of each mitonucleotype (e.g. *AA_1_*, *AA_2_*, *AA_3_*) which would be refuted by frequent among-replicate differences on the same diet. Of the 36 total comparisons (3 diets, 12 populations), only four were significantly different, indicating that mitonuclear replication led to repeatable fecundity. Second, we assessed whether genetic replicates responded equivalently to dietary change. Adding lipid consistently repressed fecundity, but the magnitude varied repeatably by mitonucleotype (Figure S2C, Figure S2F). Responses to EAA enrichment were more nuanced but were still overall repeatable among replicates, with most populations increasing egg laying, to the greatest extent in *BA* populations but more modestly in *AA*; and fewer populations responding to EAAs in *B* nucleotypes independent of mitochondria (Figure S2F). These analyses confirmed that (A) fecundity across varied diets was repeatable among independently replicated mitonucleotypes, and (B) dietary EAAs and lipid can cause this variation.

1. **Multi-trait phenotyping with chronic and parental diet manipulation: analysis by geographic origin.**

In the larger experiments, when multiple traits were measured in multiple feeding paradigms, we calculated Estimated Marginal Means (EMMs [12] to visualize patterns of DMN variation, and repeatability among replicate mitonucleotypes. DMN variation was visually apparent for all traits (Figure 2C) We excluded development data for mitonucleotype 4 (population *AA_3_*) in the chronic paradigm from some statistical analysis (per-population ANOVA tests) because its extreme trait values complicated modelling. We do not discard this line as an outlier: rather we highlight its extreme and unique response to diet, which is self-evident without statistical modelling. In other populations, statistical models revealed DMN interactions for all traits in each feeding paradigm (GLMMs for fecundity, progeny and fertility; Cox mixed-effects models for development time), except for fecundity in the parental feeding paradigm (Table S8). For development time models, we also included interactions with offspring sex, because of reports of sex-biased mito:nuclear variation [5]. However, sex did not modify genotype-by-diet interactions (all sex interactions with diet, mitochondria, or nuclear background: p>0.05).

Chronic lipid feeding was deleterious for all traits, but mitonuclear genotype determined magnitude; chronic EAA feeding promoted fecundity but, surprisingly, reduced fertility (Figure 2C). Consequently, progeny counts on EAA did not exceed counts on control diet, suggesting that fitness was not enhanced by EAAs. The magnitude of the fecundity benefits and fertility costs were again mitonucleotype-dependent. In *AB, BA* and *BB* populations, chronic lipid feeding reduced fertility most strongly, whereas effects of chronic EAA feeding were mixed. However in *AA* populations uniquely, progeny count after chronic EAA feeding was even lower than after chronic lipid enrichment, to near lethality in *AA_3_* (Figure S6C). This sensitivity was absent in both the *BA* and *AB* populations (Figure S6C): Since switching either mitochondria or nuclei was sufficient for rescue, the data indicate a mitonuclear effect. Mitonuclear incompatibility is widely reported [13], as are DMN effects on physiology and life-history [1–5],: the present data now indicate that mitonuclear incompatibility can be diet-dependent, under nutrient-enriched conditions that we had expected to promote fitness.

DMN variation was also apparent in the parental feeding paradigm, albeit less pronounced than after chronic feeding. Lipid was less universally toxic. *AA* flies even exhibited a benefit of parental lipid feeding, developing on average one day earlier (Figure S6D), but again this was not evident in *BA* or *AB* flies (Figure S6D), confirming another mitonucleotype-specific effect.

To estimate repeatability for all traits, we used the same approach as in our initial fecundity experiments (Supplementary Text 2), and found among-replicate repeatability both within each diet and in response to nutrient enrichment, for all traits (Figure S8).

1. **AIC and r^2^ calculations**

For orthogonal tests of the importance of DMN terms, on top of effect size calculations, we asked if alternative models excluding DMN terms were better descriptors of the data. We fit a structured series of models for each trait, systematically including or eliminating diet, mitotype and nucleotype, and their interactions. Interactions with offspring sex were also included for development models. We calculated Akaike Weights (Table S9, Table S10), which evaluate the relative performance of a set of models. For all traits but one (fecundity, in parental feeding paradigm), Akaike Weights showed that the best-performing models included DMN interaction terms. For development time in each feeding paradigm, higher interactions between sex and diet, mitotype and nucleotype were generally not favored, consistent with the suggestion from effect size calculations that offspring sex was not a major modulator of genetic and dietary effects in these flies.

We also calculated variance explained (r^2^) by GLMMs (fecundity, progeny, fertility: r^2^ cannot be calculated for Cox models of development time), to evaluate each model’s capacity to statistically predict phenotype. GLMMs favored by Akaike Weights explained between 65% and 95% of variance (Table S9), suggesting that most sources of variation in our flies were accounted for by our models.

1. Mossman JA, Biancani LM, Zhu C-T, Rand DM. Mitonuclear Epistasis for Development Time and Its Modification by Diet in Drosophila. Genetics. 2016;203: 463–484. doi:10.1534/genetics.116.187286

2. Rand DM, Mossman JA, Zhu L, Biancani LM, Ge JY. Mitonuclear epistasis, genotype-by-environment interactions, and personalized genomics of complex traits in Drosophila: Mitonuclear G x G x E. Iubmb Life. 2018;70: 1275–1288. doi:10.1002/iub.1954

3. Zhu C-T, Ingelmo P, Rand DM. G×G×E for Lifespan in Drosophila: Mitochondrial, Nuclear, and Dietary Interactions that Modify Longevity. PLoS Genetics. 2014;10. doi:10.1371/journal.pgen.1004354

4. Montooth KL, Dhawanjewar AS, Meiklejohn CD. Temperature-sensitive reproduction and the physiological and evolutionary potential for Mother’s Curse. Integr Comp Biol. 2019;59: 890–899. doi:10.1093/icb/icz091

5. Camus MF, O’Leary M, Reuter M, Lane N. Impact of mitonuclear interactions on life-history responses to diet. Philosophical Transactions Royal Soc B. 2020;375: 20190416. doi:10.1098/rstb.2019.0416

6. Grandison RC, Piper MD, Partridge L. Amino-acid imbalance explains extension of lifespan by dietary restriction in Drosophila. Nature. 2009;462: 1061–1064. doi:10.1038/nature08619

7. Emran S, Yang M, He X, Zandveld J, Piper MD. Target of rapamycin signalling mediates the lifespan-extending effects of dietary restriction by essential amino acid alteration. Aging. 2014;6: 390–8.

8. Dobson AJ, He X, Blanc E, Bolukbasi E, Feseha Y, Yang M, et al. Tissue-specific transcriptome profiling of Drosophila reveals roles for GATA transcription factors in longevity by dietary restriction. npj Aging and Mechanisms of Disease. 2018;4. doi:10.1038/s41514-018-0024-4

9. Hariri N, Thibault L. High-fat diet-induced obesity in animal models. Nutr Res Rev. 2010;23: 270–99. doi:10.1017/s0954422410000168

10. Heymsfield SB, Wadden TA. Mechanisms, Pathophysiology, and Management of Obesity. New Engl J Med. 2017;376: 254–266. doi:10.1056/nejmra1514009

11. Simpson SJ, Raubenheimer D. The Nature of Nutrition. 2012. doi:10.1515/9781400842803

12. Searle SR, Speed FM, Milliken GA. Population Marginal Means in the Linear Model: An Alternative to Least Squares Means. Am Statistician. 1980;34: 216–221. doi:10.1080/00031305.1980.10483031

13. Hill GE, Havird JC, Sloan DB, Burton RS, Greening C, Dowling DK. Assessing the fitness consequences of mitonuclear interactions in natural populations. Biol Rev. 2018;94: 1089–1104. doi:10.1111/brv.12493
