# Supplementary figures and images for: Mitonuclear interactions shape both direct and parental effects of diet on fitness and involve a SNP in mitoribosomal 16s rRNA
Source: PLoS Biol. 2023 Aug 21;21(8):e3002218. doi: 10.1371/journal.pbio.3002218 (PMC10441796; doi:10.1371/journal.pbio.3002218)

Figure S1

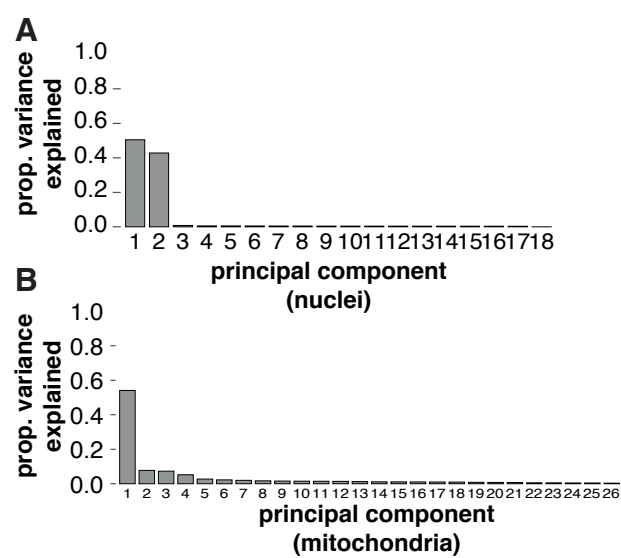

Supplement: S1 Fig — Barplots show variance explained by (A) nuclear SNPs and (B) mitochondrial SNPs. Data underlying the graphs shown in the figure can be found in S16 and S17 Tables. (PDF) [file pbio.3002218.s002.pdf]

### Figure S3

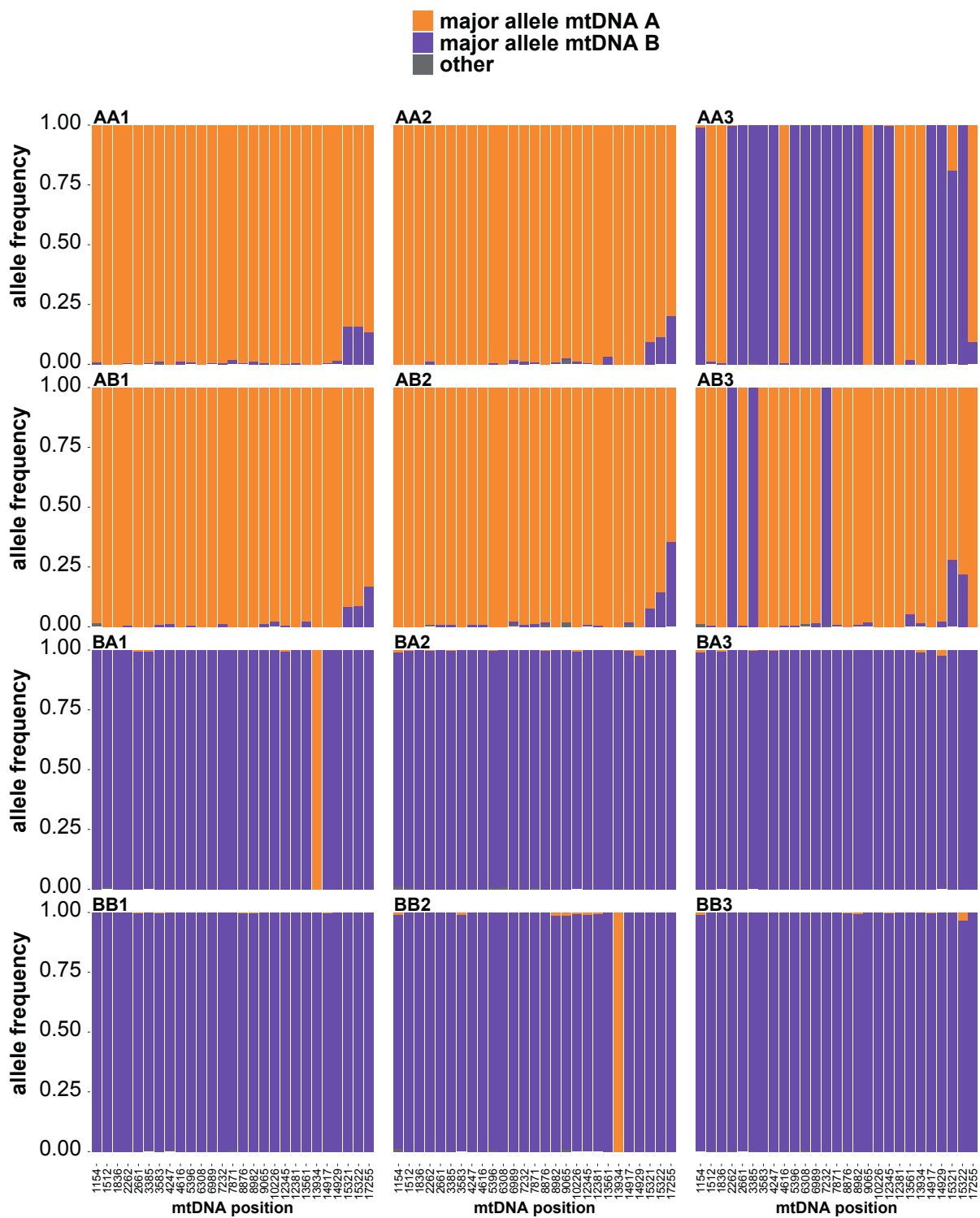

Supplement: S3 Fig — In total, 146 SNPs were observed within the 12 populations, of which 27 were significantly differentiated between populations with mtDNAs of different origins. Significant allele frequency differences were assessed by Fisher’s exact test (FDR < 0.001). Stacked barplots show allele frequencies at each locus, per population. Data underlying the graphs shown in the figure can be found in S21 Table. (PDF) [file pbio.3002218.s004.pdf]

Figure S4

protein-coding gene

tRNA

rRNA

SNP

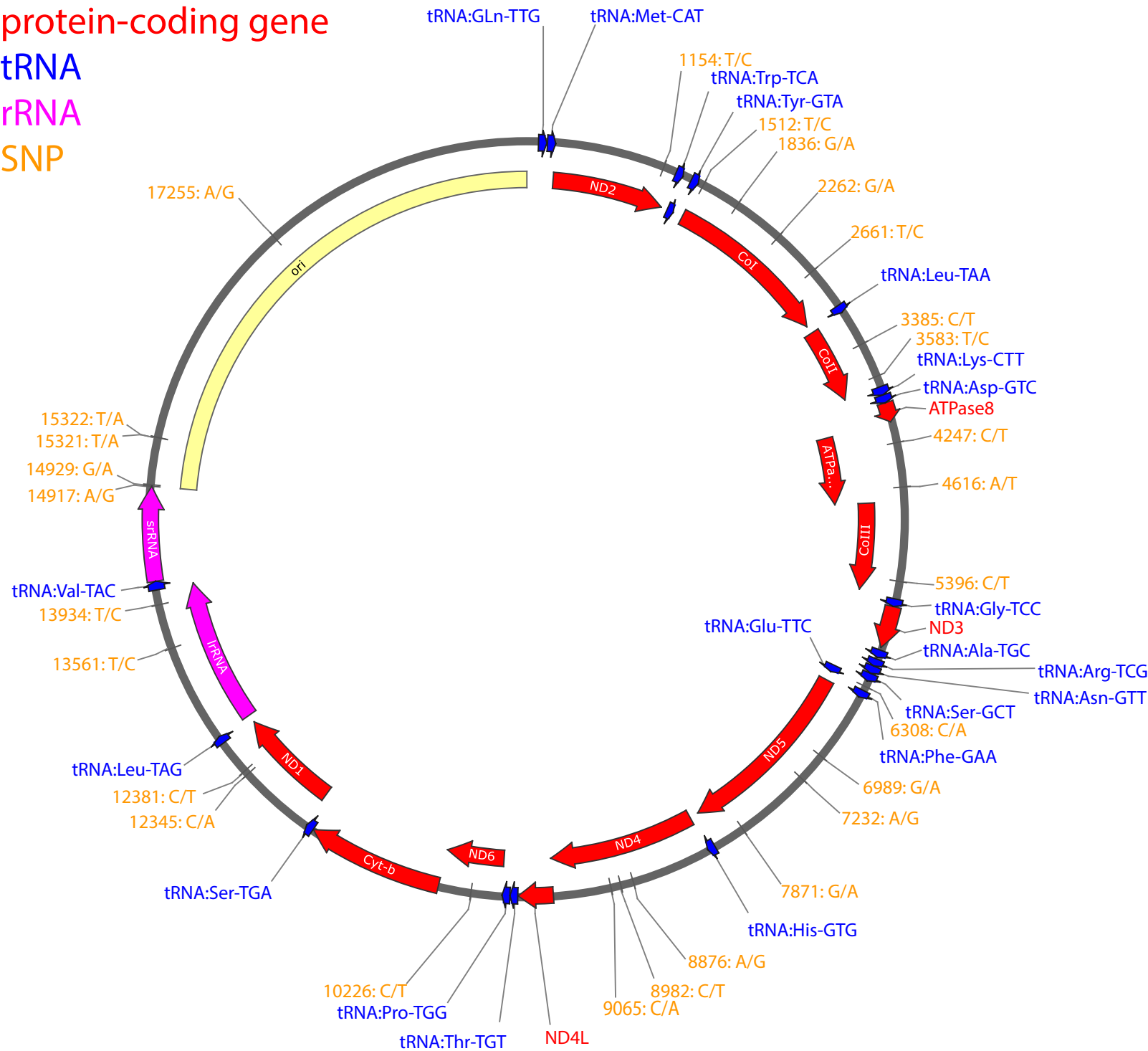

Supplement: S4 Fig — Red shows protein-coding genes; blue shows tRNAs; and purple shows rRNAs. Positions of significantly differentiated SNPs shown in orange, with position and alleles. (PDF) [file pbio.3002218.s005.pdf]

Figure S5

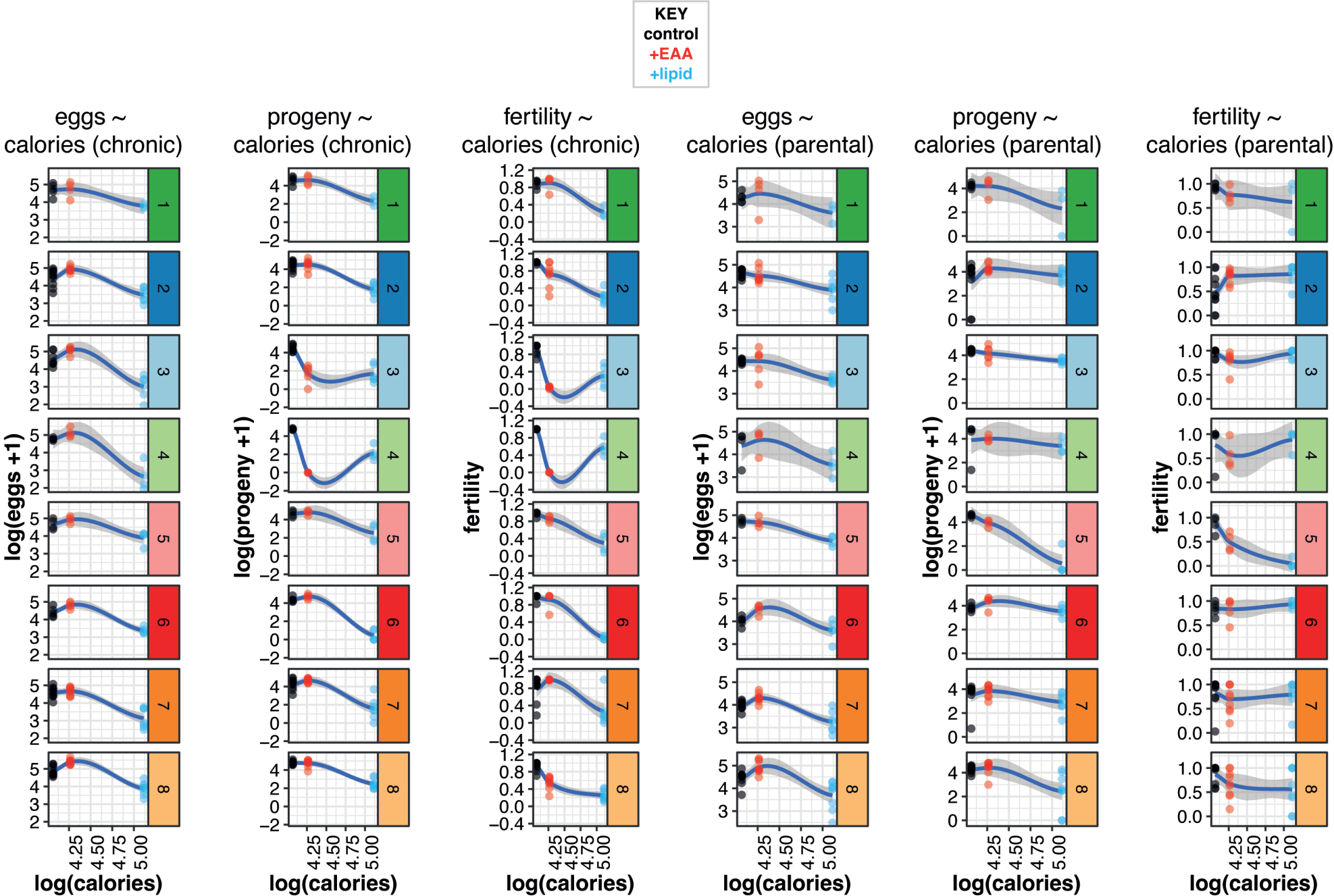

Supplement: S5 Fig — Trait and feeding paradigm indicated above each panel of plots. Within each panel, scatterplots show trait values at each caloric level, with rows for each mitonucleogenotype. Diet indicated by color. Populations show smoothed spline through points. Egg and progeny counts are presented x+1 to enable plotting log values. Trait values do not linearly correlate with calories; therefore, caloric content is no more informative than modeling diet as an unordered factor. Data underlying the graphs shown in the figure can be found in S22 Table. (PDF) [file pbio.3002218.s006.pdf]

Figure S6

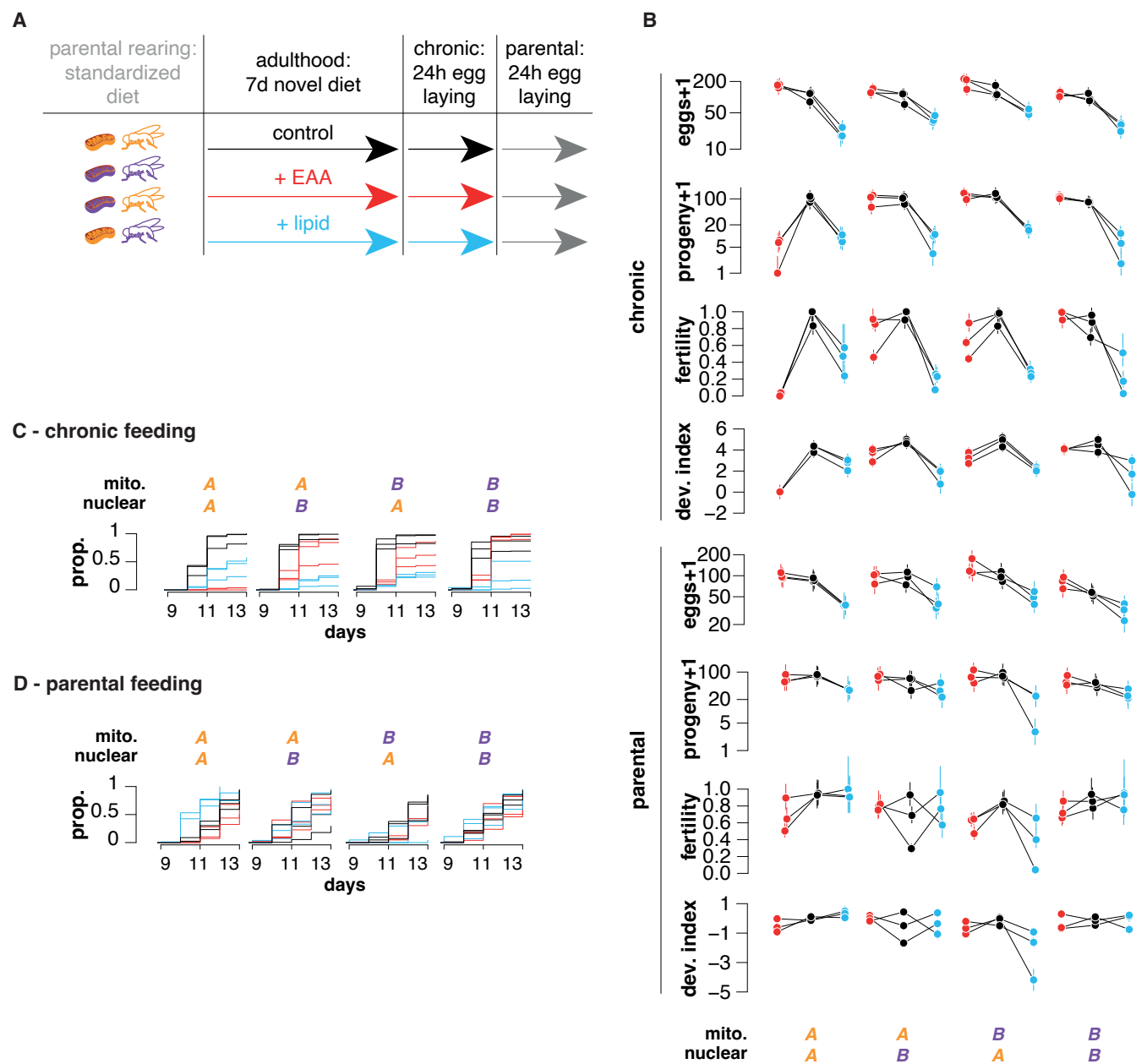

Supplement: S6 Fig — (A) Key and experimental design. Flies were reared from egg to adult on rearing food and allocated at random to experimental media 6–48 hours after eclosion, at a density of 5 of each sex per vial. After 7 days, flies laid eggs on fresh food for 24 hours, followed by a further 24 hours on standardized rearing medium. (B) Mitonuclear variation in response to chronic and parental changes in nutrition. Panels show EMMs (±95% CI) for trait indicated on y-axis. Feeding paradigm and mitonuclear variation are indicated at the top of the plot. Colors encode diet as per panel A, egg and progeny counts are presented as x+1 to enable plotting on log scale. Development index shows EMMs for Cox mixed-effects models of proportion eclosed over time, excluding sex from plot. Development data are plotted in full as Kaplan–Meier plots in panels (C) and (D). Note the exclusion of EMMs for development of genotype AA3 in chronic feeding: EAA lethality prevented meaningful estimation. (C, D) Kaplan–Meier plots of development for the indicated feeding paradigms. Plots show proportion eclosed over time. Colors encode diet as per panel (A). Data underlying the graphs shown in the figure can be found in S23 and S24 Tables. (PDF) [file pbio.3002218.s007.pdf]

PC2

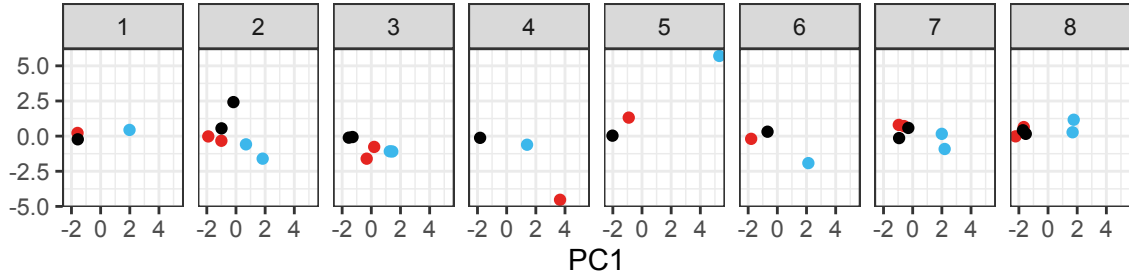

Supplement: S7 Fig — PCA shows ordination of populations according to mitogenotype, nucleogenotype, and diet. Results shown from PCA of scaled and mean-centered EMMs, split by facets per genotype, with mitonucleogenotype split by rows. Data underlying the graphs shown in the figure can be found in S25 Table. (PDF) [file pbio.3002218.s008.pdf]

### Figure S8

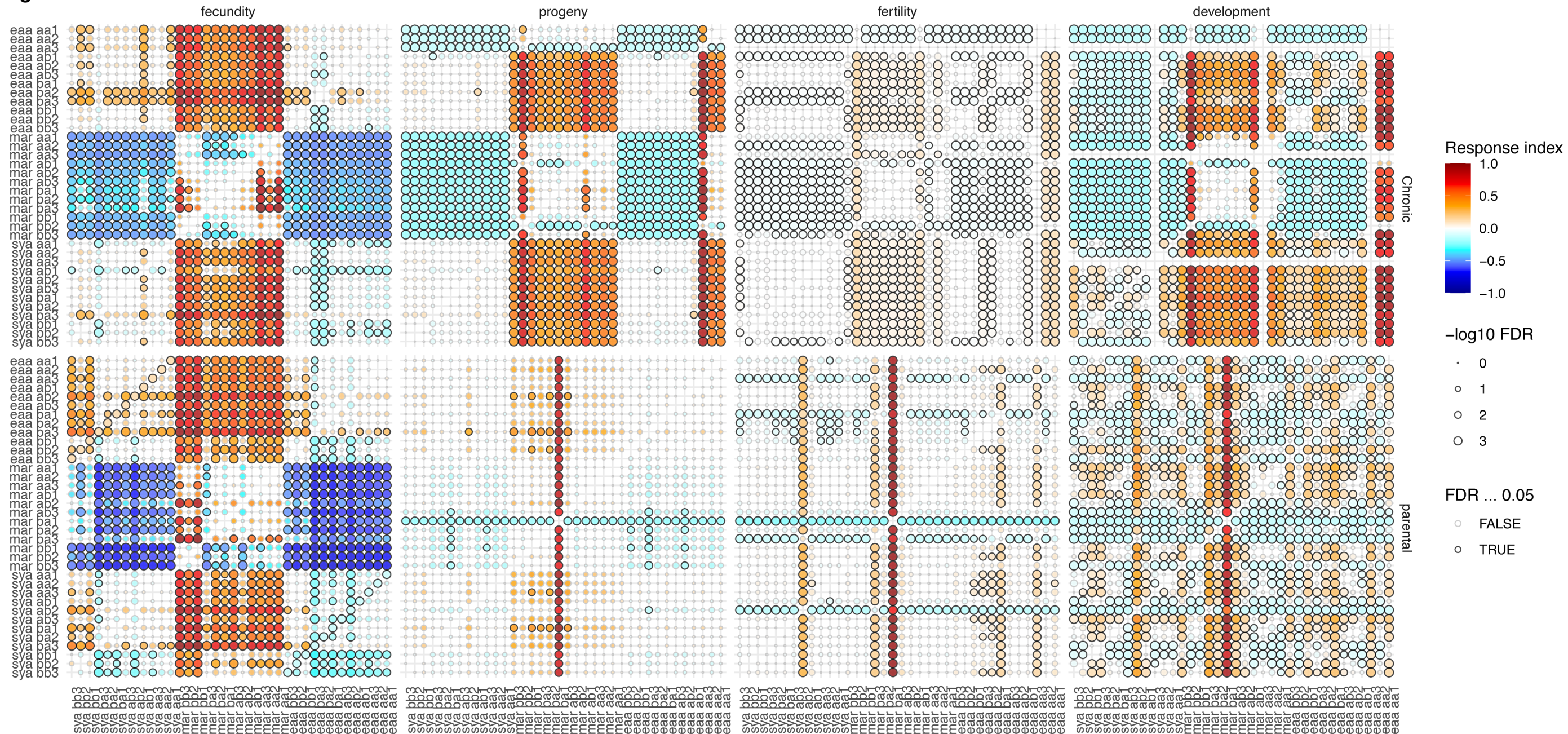

Supplement: S8 Fig — Bubble plot shows response index—signed, logged, absolute fold-change in specified comparisons of EMMs—with point size scaled to indicate probability of observed difference (−log10 FDR), and border opacity indicating threshold of statistical significance (FDR ≤ 0.05). Fold-change calculated for conditions on y-axis relative to conditions on x-axis, e.g., bottom-right cluster of points shows increase on EAA-enriched media relative to control. Points along diagonal show comparisons within replicate genotypes on the same diet, with few significant differences among replicate genotypes. In response to lipid enrichment, the same changes were always evident in replicate genotypes, and in response to EAA enrichment, similar changes were evident in some replicates. Boxes indicate comparisons within replicate populations on the same diet. Data underlying the graphs shown in the figure can be found in S26 Table. (PDF) [file pbio.3002218.s009.pdf]
