# Supplementary material for: Mitonuclear interactions shape both direct and parental effects of diet on fitness and involve a SNP in mitoribosomal 16s rRNA
Source: PLoS Biol. 2023 Aug 21;21(8):e3002218. doi: 10.1371/journal.pbio.3002218 (PMC10441796; doi:10.1371/journal.pbio.3002218)
Supplement: S2 Fig — (A) Reproductive manipulation by enriching fly medium with plant-based lipid. Egg laying by wild-type Benin flies (the ancestral population from which B populations were derived) after 7 days feeding on control medium (10% yeast, 5% sugar) and 15% added plant-based lipid source (margarine). After 1 week of feeding, flies were switched to development medium for egg-laying assay to ensure that any effects resulted from physiological impacts of manipulation and not differences in preference for oviposition on the food. Boxplots show medians, first and third quartiles, and fifth and 95th percentiles. Two-sample t test t = 1.98, df = 16, p = 0.03. Data shown per fly. (B) Key and experimental design. Flies were reared from egg to adult on rearing food and allocated at random to experimental media 6–48 hours after eclosion, at a density of 5 of each sex per vial. After 7 days, flies laid eggs on fresh food for 24 hours. (C) Mitonuclear variation in fecundity response to nutrient enrichment. Plot shows eggs laid in vial of 5 females and 5 males over 24 hours. Boxplots show medians, first and third quartiles, and fifth and 95th percentiles. Points to left of each box show raw data. Connected points to right of each box show estimated marginal means (EMMs) with 90% confidence intervals. Data shown per vial (5 females + 5 males). Egg counts are presented as x+1 to enable plotting log values. (D) Fecundity does not correlate caloric content of experimental media. Scatterplots show eggs at each caloric level, with facets per each combination of mitochondrial (columns) and nuclear (rows) genotype. Diet indicated by color. Populations show smoothed spline through points. Egg counts are presented x+1 to enable plotting log values. Trait values do not linearly correlate with calories; therefore, caloric content is no more informative than modeling diet as an unordered factor. (E) Technical repeatability of diet:mito:nuclear effect between replicate experiments. Each point shows mean [file pbio.3002218.s003.pdf]

Figure S2

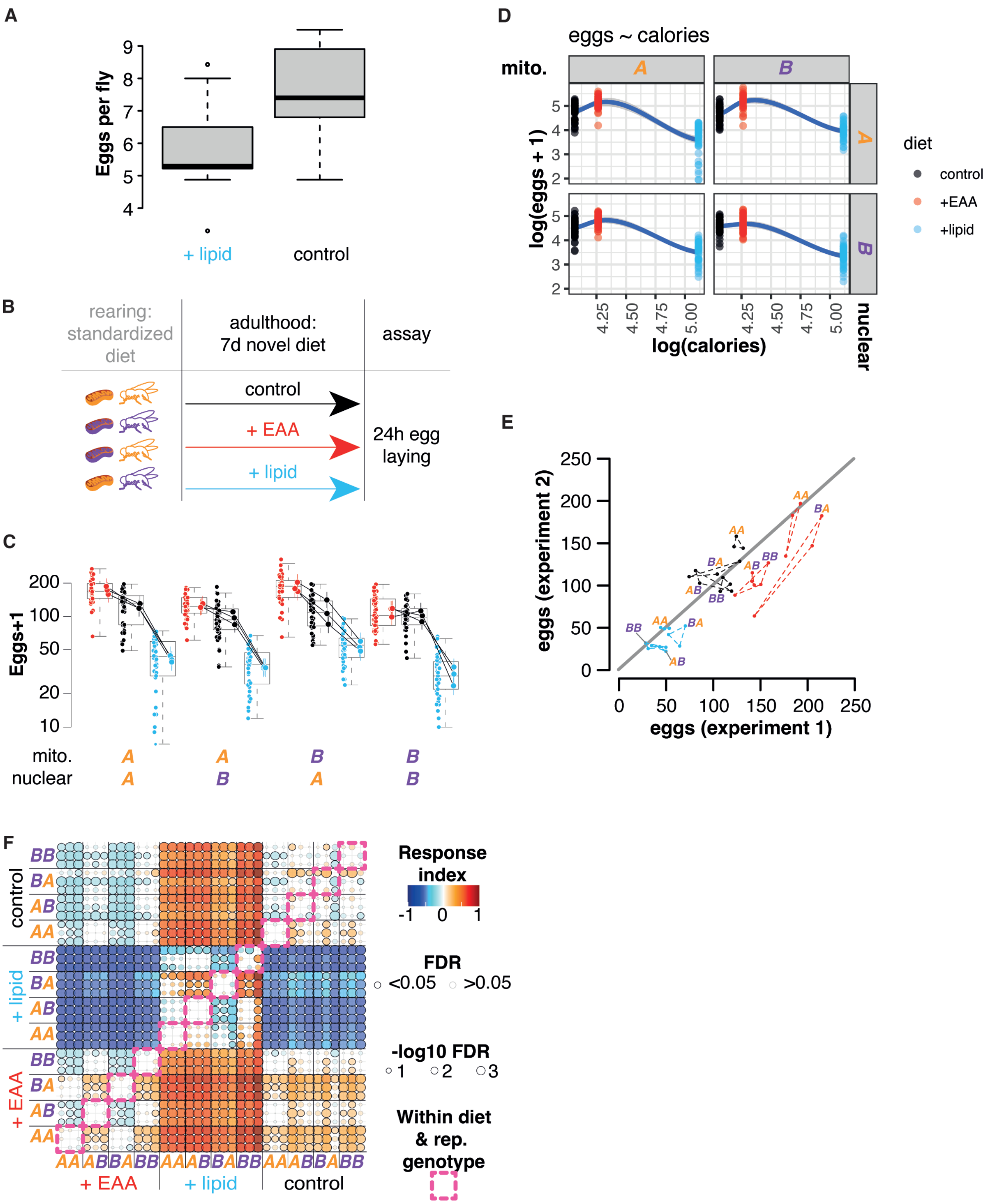

A
Eggs per fly
+ lipid
control
B
rearing: standardized diet
adulthood: 7d novel diet
assay
control
+ EAA
+ lipid
24h egg laying
C
Eggs+1
mito.
nuclear
D
eggs ~ calories
mito.
log(eggs + 1)
log(calories)
nuclear
diet
control
+EAA
+lipid
E
eggs (experiment 2)
eggs (experiment 1)
F
control
+ lipid
+ EAA
Response index
FDR
<0.05
>0.05
-log10 FDR
Within diet & rep. genotype
