# Supplementary material for: Mitonuclear interactions shape both direct and parental effects of diet on fitness and involve a SNP in mitoribosomal 16s rRNA
Source: PLoS Biol. 2023 Aug 21;21(8):e3002218. doi: 10.1371/journal.pbio.3002218 (PMC10441796; doi:10.1371/journal.pbio.3002218)
Supplement: Attachment — Submitted filename: response to reviews.pdf [file pbio.3002218.s038.pdf]

We thank the reviewers for their insightful and constructive comments, which have inspired revisions that greatly improved the paper. We have restructured our results fundamentally and changed emphases and sequence of results. Our specific actions were:

- Emphasised the novel parental diet:mito:nuclear effect on offspring health.
- Moved supplementary figures into main figures to make clear the genetics of the lines, moved network and heatmap figures into this figure, reworked presentation of admixture analysis.
- Generated new figure 3 showing effect sizes systematically.
- Reordered and streamlined the results, emphasising the relationship between mtDNA sequence and phenotype, obviating need to refer to geographic origins of mitotypes and nucleotypes.
- Moved materials & methods into main body.
- Reworked Figure 2 to incorporate all aspects of useful genetic information and its relationship to phenotype.
- Abstract reworked to emphasise novel aspects of the study
- Mitochondrial genome map added.
- Added text to clarify that lack of mitotype nesting within nucleotype indicates a lack of incompatibility on the rearing diet.
- Improved explanation of admixture analysis.
- Added text explaining the unique mitotype of mitonucleotype AA3, and associated unique phenotype.
- Added text to results to discuss in more depth that the intermediate mitotype in AA3 shows lethality.
- Clarified in methods when data are per-vial not per-fly.
- Toned down discussion of repeatability.
- Confirmed whether other studies use equally specific diet modifications.
- Added text to explain what can or cannot be concluded about major alleles and haplotypes from pool-seq data.
- Added text to highlight that the study uses populations of flies, not isolines.
- Added text to clarify that qualitatively different effects can also be statistically significant in quantitative terms.
- Removed term "genetic replacement".
- Clarified that "because introgression maintained variation and therefore phenotypic variation could be assoc with sequence data", we mean that we could not see evidence of drift, and so the panel appears to be a suitable mapping population.
- Clarified that "unprecedented" meant "unprecedented effect size".

We enclose a point-by-point response to reviewers below (reviewers' comments in red)

####Reviewer 1####

Page 15, line 75 - This sentence confused me - "This approach is expected to dilute with the paternal nucleotype and before eventually purging the F0 mother's nucleotype, while retaining the F0 mother's mitotype." I think the "and" is erroneous.
We have reworded this sentence.

Page 17, line 118 - 120 - This may be a naive question. To ensure genetic consistency you use the same parents in each paradigm. These parents spend one week on experimental media (chronic paradigm) and then are allowed to lay eggs for 24 hours. They are then switched to a universal medium for another 24h of egg laying (parental paradigm). Is 24 hours on the universal medium sufficient to remove/minimize the effects of the experimental media? Could diet effects from the experimental media persist into egg laying while on the universal medium?
This is correct. Our goal was to assess mitonuclear variation in those persisting effects, indeed this is why we switched diet as we did. Eggs laid on experimental media have not experienced dietary variation, rather their parents did. Thus any effects of diet that we see in this generation are exclusively persistent effects of parental diet. Since we see effects of diet in this paradigm, we conclude that the effects of parental diet do indeed persist into offspring over this 24h period - as we hoped and expected when designing our experimental manipulation. Furthermore, since we can see diet:mitonucleotype effects, this persistent effect of parental diet is subject to mitonuclear variation.

In my previous review, I asked about co-adaptation between the mitochondrial and nuclear genome. The response to reviewers mentions that a commentary on co-evolution between the mitochondrial/nuclear genomes was added on lines 289 - 297. These lines do not mention co-evolution/adaptation, and I could not find any commentary in the rest of the discussion. I'm curious whether the referenced text was not added to the version we have.
This paragraph is at lines 481-496 of the resubmission. We can see that our phrasing around matching and mis-matching in the previous draft may have been unclear. We have therefore ensured that it now opens with unambiguous phrasing, to make the focus of the paragraph clear.

###Reviewer 3:###

Major comments:
The main text should more clearly describe the underlying genetics... The main text has no methods and the supplement is extensive, making it hard for the reader to find the key methodological and results needed to understand the main text.
We have moved materials and methods from supplementary materials into the main text. We have also moved a figure describing the introgression protocol from supplementary materials to the main figures (now Figure 1), and incorporated material from other figures to maximise clarity.

One could imagine a more streamlined presentation of the data where the mtDNA genotyping is presented first with all subsequent analysis presented using the sequence-based mitonuclear genotypes rather than the AA,AB,BA,BB categorization.

We have restructured the paper in light of this comment. We have now brought all genomic analyses into Figure 1, phenotyping using the sequenced-based mitonucleotypes into Figure 2, and effect size calculations into Figure 3.

Was the Australia source stock/population segregating mtDNA haplotypes that are very similar to Benin? If so, why?

We would like to highlight that we worked with populations, not isolines. Therefore (A) our genotyping reveals major alleles, and (B) the results in figure 1G (formerly 2A) are major alleles at each position, not haplotypes. We have taken efforts to highlight this difference in the figure legends and in the results section. Our crossing schemes were rigorous and fastidious and so it seems that these alleles, derived from the ancestral stock, rose to high frequency in this population. However, a strength of our approach is that we use our sequencing data to assign populations to mitonucleotypes, and so the source of the variation does not affect our conclusions: the presence of variation in the ancestral population is not relevant to our findings. Indeed it is precisely because of our sequencing approach that we are able to show that population AA3 is an outlier both in terms of its mitotype (Figure 1) and phenotype (Figure 2).

Was a single female sampled to provide the mtDNA for each replicate mitochondrial-introgression line? If not, do you have evidence that there are not segregating mtDNAs within each replicate line?...was a single female used to establish the two mtDNA's in the study?...Are the populations segregating mtDNA variation?...This should be more clearly explained in the results (or the methods provided before the results).

We have moved materials and methods into the main text to clarify, and we have reworked Figure 1 extensively to make the genetics clearer.

Our study used multiple females from an outbred starting population, to enable variation to segregate in mitochondrial genomes. This was by design. Figure S3 makes clear that variation segregates in the population's mitochondria, although major alleles are at very high frequency (see Results, and Supplementary Table S3).

We did not sample single females, because the populations were not isolines. It was for this reason that we used pool-seq. To make this clear throughout, we have systematically replaced the word "line" with "population". We have also added text at lines to make as clear as possible that the populations were not isogenic.

However, once the reader gets to figure 2, it makes it seem inappropriate to include AA3 as an "A" mitotype in the analyses done in figure 1.

In light of this feedback we have revised the emphasis of our analysis extensively. As described above, a strength of our paper is that we use genome sequencing to model phenotypes. The geographic origin of genomes is irrelevant with the sequence in hand. We have therefore revised the order of the results, emphasising genotyping (Figure 1), followed by our sequence-led modelling of phenotypes over mitonucleotypes (Figure 2). We have moved the analysis by geographic origin to supplementary materials.

We agree that mitochondria from population AA3 are intermediate between other A mitochondria and B mitochondria. We have moved network analysis and clustering

to Figure 1F and 1G to make this point clear. The intermediate position of this mitotype/population is highlighted in-text.

Why is C included in the paper at all and what is the relationship between mitotypes in B and C populations?
We have included sequencing results from C populations, before their dismissal, because (a) they were a part of previous studies using the same panel of populations (e.g. PMID: 32053259) and therefore (b) principles of open science obliged us to present the full breadth of our sequencing data. The text explains our rationale for excluding the Canadian populations from phenotyping – we anticipated little gain from their inclusion, and their exclusion made the 3-diet experiment tractable. Having excluded the populations from phenotyping, the relationship between C mitotypes and others are beyond the scope of our study, however the sequence data will be freely publicly available for further analysis if this is of interest.

In figure S1, the A nuclear genome has more admixture from C and the A mitotype appears to have C mtDNAs. Is this from past contamination of a C/B-like mtDNA into the A source population? Is the remnant C nuclear contribution to the A nuclear introgression lines what randomly remains after introgression or is it potentially selected for by the presence of the B/C mtDNA? Is this from evolutionary history in the source populations in the lab or from the history of introgression during the experiment? I write B/C-like because it was not clear from the combination of information from fig 2 sequence alignment and the fig S1 structure analyses what is the source of the AA3 mtDNA... Figure S1 assigns the mtDNA in AA3 to Canada, but the figure 2 alignment makes the mtDNA appear to be very similar to Benin... Does the Benin/Canada-like mitotype(s) segregate in the "A" stocks used for the Canadian and Australian cytotype?

We feel these comments may reflect a lack of clarity in our previous explanation of what the admixture analysis showed. We have worked on the text and updated figures to clarify.

Admixture modelling shows similarities in variants among populations, without inference on origin. The populations are grouped to a number of user-defined clusters - the analysis does not show a proportion of genome X originating from genome Y. In light of these comments, we have re-examined the presentation and writeup of our admixture analyses (Figure 1) in depth. We have added text to results at lines 112-116 to make this more clear. We have also re-coloured the figures (now Figures 1C and 1E), since those in the previous draft gave the appearance of "variation from population A/B/C" when it should more accurately have been termed "cluster most dominant in population(s) A/B/C" - to simplify we have re-coded simply as clusters 1/2/3, which bear no intrinsic similarity to ancestral state. We appreciate that the colouring of these figures in the previous draft may have been confusing, and we thank the referee for comments that brought this to our attention.

As we now state in-text (lines 120-122 and 132-135), the admixture analyses complement the PCA analyses, showing similarity/differentiation among the populations. We have made sure to cite the method (Bradburd et al, 2016) extensively throughout this section for a full explanation of the method. We had the method assign to three clusters to reflect our use of three ancestral populations, but we could equally have used any number of clusters (see Bradburd et al, 2016).

It is beyond the scope of this study to characterise further the relationship between C mitotypes and A or B, since we did not phenotype C populations. However one might expect that AA3 was similar to C mitotypes, since AA3 is similar to B mitotypes, and B were similar to C.

The major alleles of all segregating SNPs are presented for each mitotype in Figure 2A. As we acknowledge in the text and make clear in figures, the AA3 mitotype is intermediate between B mitotypes, and the AA1/2 mitotypes. A strength of our design is that our sequencing allows us account for this variation. Consequently we do not need to worry about the frequency of variants segregating in ancestral populations: we have characterised it and incorporated into our models, at which point all that matters is the sequence and its relationship to phenotype, not the ancestral state.

Maybe a mtDNA haplotype map might help here?
We do not know haplotypes for certain, because SNPs were called using Pool-Seq. Instead we have moved all genomic analyses into Figure 1, and we have added a map of the mitochondrial genome showing genes and the SNPs of interest (Figure S4). We have also added text into results stating explicitly that we do not know haplotypes for sure.

Were these cytotypes cleared of or checked for the presence of wolbachia?
Yes. This information is included in the materials and methods, now moved into main text. The information can now be found at lines 566-567.

As pointed out by reviewer 2 from review commons, this adds to a growing number of studies in flies that demonstrate mito-nuc-diet interactions as an important source of variation for phenotypes, including reproduction, development and survival. A strength of the design is the ability to contrast chronic versus parental diet effect, which is novel, particularly how this implicates mito-nuclear-diet interactions as important for provisioning gametes. This could be discussed more.
In rewriting the manuscript we have attempted to emphasise the novelty of the parental effects as much as possible, including a new title and abstract. The results have now been rewritten, and in doing so we have attempted to emphasise the novelty of parental effects whenever possible.

To treat this three-way interaction as "unprecedented" incorrectly represents the literature.
We realise that in a push for brevity, our phrasing here did not correctly convey our intention. We thank the reviewer for highlighting. We have clarified that "unprecedented" meant "unprecedented effect size" – i.e. equal to that of diet, and qualitatively determining survival or lethality (which are unprecedented). We have amended the text accordingly throughout.

…the paper ultimately misses the opportunity to focus on the interesting biology of the candidate SNP that is identified...Other studies in flies...have shown diet can modify mito-nuclear incompatibilities that cause sterility and impact egg-to-adult survival...The diet manipulations in other studies are no less specific than those used in this study, just different... The paper could be more focused on...how a

mitochondrial SNP putatively affecting mitochondrial protein translation may interact with nuclear genome and the environment to affect gametogenesis and development. There is supporting research in this area... Line 145-146, I fail to see the distinction being made here between some of the studies you cite and this study. Some of the studies that you cite provide evidence that mitonuclear incompatibilities interact with diet to impact reproductive traits, which is the same fitness trait in this study. Other studies (I think not cited) have shown effects of diet on egg-to-adult survival in mitonuclear genotypes.

We have checked that we cite the landmark Mossman et al (2016) paper and added a short paragraph to the discussion to ensure that their egg-to-adult viability data are referenced. We hope that we interpret correctly that their data do not show any line experiencing diet-dependent lethality in the same way as the flies in our study.

Previous studies manipulated dietary yeast and sugar. In our study, nutrition was manipulated by adding specific classes of nutrients to the diet and measuring the resulting diet-mito-nuclear variation. We have checked the literature that we are aware of, and we have not found any preceding diet-mito-nuclear studies that have added specific nutrients to fly media.

We have updated our references to previous work potentially related to mitochondrial translation, and added citations to ensure that the supporting literature is recognised. We hope this is satisfactory and we thank the referee for pointing out this oversight.

The discussion is framed around regulatory versus coding effects. lrRNA SNPs are not typically described as regulatory. The lrRNA function in the ribosome to catalyze protein synthesis. SNPs in these genes have the potential to affect the function of the molecule that they encode.
We agree that the previous wording was not sufficiently precise. We have now opted for "non-protein-coding", throughout.

The framing with the omnigenetic model also seems out of place. A single mtDNA SNP with detectable effects on fitness is not exactly the type of variation that the omnigenetic model describes.
We interpret our results in the context of the omnigenic model in the discussion, as a point of debate. Our interpretation is that the omnigenic model suggests that alleles in a subset of genes will determine the output of variation in other parts of the genome, and that this is a useful framework for discussing mito-nuclear epistasis. We note that other reviewers agree with our interpretation, and we feel that our view is consistent with a recent landmark review (PMID: 34964900). Overall we welcome debate in the field as healthy. We therefore feel our discussion of the omnigenic model is a worthwhile inclusion, that may more closely integrate the mito-nuclear field with an important new development in quantitative genetics.

Although, clearly any GxGxE interaction indicates variation that does not have deterministic effects on fitness and thus may be a source of complex genotype-phenotype relationships... The questions in the abstract are not clear. I do not know what is meant by: "Are these "mitonuclear" effects deterministic with regard to optimal nutrition?" Very few genetic effects are deterministic; GxGxE variation is by nature not particularly deterministic, as they are genetic effects that are modifiable by

environmental context... I fail to understand the repeatability = deterministic argument and this seems like a contrived framing of the introduction.

It is helpful to have this view of our framing of results. When writing the manuscript our starting position was that interaction effects are complex and that outcomes may be hard to predict, but that this does not mean they are not deterministic. Rather we think that the interactions can determine trait values, though we would agree that the interactions would not make traits easy to predict. It was for this reason that we focussed on effect sizes (ie. how important are DMN interactions for phenotype) and the high repeatability among replicate populations (ie. is there less variation between replicate populations than among distinct populations). We have toned down our discussion of determinism throughout.

Or is the question whether mitonuclear genotype determine optimal nutrition, which I also find to be an odd question.

Personalised genomics has not succeeded in finding ways to personalise diet to individual need. This could be due to gaps in our understanding of the genetic basis of variation in response to diet, in which genetic variation in optimal diet is implicit. We feel mito-nuclear effects may be a major cause of missing heritability in response to diet, because epistasis is thought to be a cause of missing heritability, with mito-nuclear epistasis emerging as a major form of epistasis, and mitochondria are themselves a major hub of metabolism. It was for this reason that we calculated effect sizes, AIC values and r^2 values, for a proxy of how much variation is explained by diet-mito-nuclear terms compared to other interactions with diet, which indicate how sources of genetic variation change the response to dietary variation.

It was not clear what is meant by "the majority of mitotype diversity was represented by populations bearing only Australian or Beninese mitochondria." Figure S1 did not clarify this for me.

The PCA indicates that the majority (62%) of mtDNA diversity was represented on two PCs. On the first PC, the Canadian mitotypes were indistinguishable from Beninese. On the second PC, the Canadian mitotypes were indistinguishable from Australian. On this basis, we did not think much could be gained by including the Canadian populations, since the major axes of mtDNA variation were represented by the other two populations.

What do you think is the source of the Australian population having a Benin (or Canada?)-like mtDNA?

We suggest that this may be a misapprehension based on the previous presentation of our admixture analysis. As written above, the AA3 population has neither a typical Australian nor Beninese mitotype - it is intermediate. Again it is possible for some replicate populations to be naturally more or less similar purely because of natural variation that has risen to high frequency in a given population.

Ultimately the source of a given mitotype does not matter, since we account for among-population differences precisely by sequencing the mitotypes, which is a strength of our analysis and to date a unique approach.

Line 129-130, it is unclear whether diet is being considered as a categorical factor with caloric density as a co-factor. Are any of the diet modifications isocaloric?

In all analyses, diet was modelled as an unordered factor. This information is included in the materials and methods, in results at lines 250-251, and also in supplementary text at lines 20-21. Figure 2A has been moved from the supplement to show clearly that the diets were not isocaloric. In the main text we state that phenotypic variation was not explained by caloric variation, and this is plotted in Figures S2 and S5.

Another statistical question is how vial is being accommodated in the analyses. I was surprised by how low the p-values are in the tables given the means and variances in the fig 1 and 2 plots. Are individual vials in the experiment being treated as random factors such that individuals are not the unit of replication?
We thank the referee for highlighting this ambiguity. Our data are per-vial, not per-fly, and therefore vial was not included as a random effect by default. To clarify, we have added text to materials and methods.

The one exception to this, was for fertility under the chronic feeding paradigm, in which we included vial as a per-observation (i.e. vial) random effect, to ameliorate overdispersion (consensus solution among leading R GLMM developers: https://r-sig-mixed-models.r-project.narkive.com/fi8Nu155/r-sig-me-quasi-binomial-family-in-lme4)

Full statistical models are given in materials and methods, now moved to the main body of text as per the referee's request.

Line 142-144, the language of genetic replacement is out of place. The experiment involves a reciprocal set of mitonuclear genotypes made through introgression and not a design where you identified a genetic effect and then did a replacement to rescue the effect. I recommend sticking to the language of epistasis (GxG) where a particular phenotype is observed only in one combination of two-locus introgression genotypes.
We thank the reviewer for pointing out this potential source of confusion. Our intention was not to insinuate genetic replacement, rather that the F0 mothers' nuclear genomes were eventually diluted away by iterative backcrossing to fathers with specified nuclear genomes. We have selected a different verb ("purge") to better reflect our meaning.

Line 197-198, I do not understand what is meant by "because introgression maintained variation and therefore phenotypic variation could be associated with preceding sequence data."
We have updated the text, now at lines 166-171. Essentially, our point was that we do not appear to see any drift while the lines were being backcrossed, and so we feel it was reasonable to sequence, continue backcrossing/introgression, and then phenotype. This is an approach shared with other mapping populations.

The statement "these mitotypes were not nested within nucleotype" indicates that the designation of AA, AB, BA, and BB is by geography, but does not reflect genotype? I was surprised by this.
Our wording was meant to reflect that we do not see evidence from our sequencing that any particular mitotype was restricted to either nucleotype (except AA3, which had its own mitotype). This suggests that on rearing media there were no incompatibilities in these populations (although these emerge on +EAA food).

Lines 211-213 sounds as if you have genotype data for each individual that was phenotyped; is this the case? Or, rather, do the replicate populations have a single mitonucleotype (one of the 8?)?
Neither: we have pool-seq data which give us the frequency of alleles per population. As we have made clear at lines 180-184, we do not know haplotypes for certain. We have, therefore, conservatively referred to major alleles on mtDNA, throughout.

Lines 215-217, Why is this described as having a qualitative effect? Was it not statistically significant quantitative effect?
The effect was statistically significant - this reflects quantitative differences. In this case the sign of change was different, giving both a quantitatively significant and qualitatively different effect. The text referred to here has been removed in our overhaul of the results, but we use the same phrase on line 317.

The main text should provide statistical evidence for a nucleotype x C/T mtDNA genotype x diet interaction to support the conclusions that you draw.
We present effect sizes, and discuss these in the main text. Including further information would require us to move all statistical tables into the main body of text, which were in supplementary materials of our original draft. The editors have indicated a preference to publish this work as a short communication, which may preclude moving all 8 requested statistical tables into the main body of text. We are happy to take editorial advice on this issue.

Figure 1. If the three replicate populations are segregating different mtDNA (as I infer from the 5,6,7,8 mitotypes results section and figure 2 and S1), then I am not sure why categorizing the replicates as having A or B mitochondria is valid.
This was an especially helpful point, echoing some we have responded to above. This comment has led us to revise our presentation extensively, going from genotyping directly to phenotyping parsed over sequence-informed mitonucleotypes (Figure 2). By this approach, the origin of the mitochondria does not matter, since we care only about mtDNA sequence.

It is hard to see how AA3 is such an outlier in the PC clustering given the data in plot 1B. From the phenotypes in 1B, the three replicate populations in the AA category behave similar to each other.
Figure 2C now presents mitonucleotype 4 (aka AA3) separately from co-originating populations. This makes clear that this mitonucleotype shows distinct responses to dietary variation, for fecundity and development. It does indeed respond similarly to diet for fecundity and progeny, and so the genetic difference highlighted in the PCA does not appear relevant for these traits. It is reasonable to expect that genetic differences between populations will not necessarily lead to corresponding phenotypic differences for all traits measured.
