# Supplementary material for: Mitonuclear interactions shape both direct and parental effects of diet on fitness and involve a SNP in mitoribosomal 16s rRNA
Source: PLoS Biol. 2023 Aug 21;21(8):e3002218. doi: 10.1371/journal.pbio.3002218 (PMC10441796; doi:10.1371/journal.pbio.3002218)
Supplement: Attachment — Submitted filename: 2023_response to reviewers.pdf [file pbio.3002218.s039.pdf]

We thank referee #3 for taking time to comment again thoroughly on our revised manuscript, we feel these changes have again made the manuscript more clear. We have implemented suggested changes where possible, and where the short article format permits. We feel these changes further enchance the manuscript. Please see specific point-by-point responses below.

I appreciate the extensive revision which has improved the presentation of and made more clear aspects of the experimental design and results. Below are my detailed comments,

1) In the response to review, the authors indicate that they have downplayed determinism given the quantitative genetic nature of the variation they are describing. However, the title uses the term "dictate," which is synonymous with and perhaps even stronger than the term determine. Also, the word "determinant" is used throughout the paper including the first and last sentences of the introduction. At a minimum, can the authors please use terms more appropriate to the type of complex genetic effects that they are describing such as "impact," "affect" or "underlie"?

We show quantitatively DMN effects on the same scale as lower-order, more-recognised sources of variation in fitness. It would not be controversial to refer to such factors, e.g. diet, as fitness determinants, yet we find diet's effect is contingent on mitonucleotype, arbitrating differences as important as enhanced fertility versus lethality. This is one of our works' main messages, which was sharpened by the first round of review, and is highlighted herein by the referee's second comment. It is important to use language that conveys these impacts.

In the title we have substituted “dictate” for “define”, which we believe strikes the right balance.

However we feel that “determinant” is an appropriate, unsensational, and precise term that is rightly in common parlance.

We are happy to accept editorial guidance on this issue.

2) Showing that mito-nuc-diet effects are on the same order of effect size is an interesting result that I think should be highlighted in the paper. This conclusion rests on the use of partial eta-squared statistics, which I understand to be SS(of focal effect) divided by the SS(error and all other factors). In Figure 3B and C, which I think are the stronger analyses for evidencing DMN effects in the study, how can so many of the factors have a partial eta-squared statistic so close to a value of one? This is particularly evident in the analysis of development. It seems to me like the statistics should sum to something on the order of 1. The other piece of information to report in the main text along with Figure 3 would be the percent of the total variation explained by the model, which would complement the point being made about equivalent relative effect sizes while also providing the reader with a sense of how much total variation is explained by diet, genetic compartments, culture/vial effects, and interactions between factors.

We thank the referee for recognising the importance of the effect size results. We separate our response to points about effect sizes and R^2 calculation:

EFFECT SIZES

Our measure of effect size is partial eta-squared. This differs from Eta-squared (ie. not partial) in that the values are calculated from test statistics. We used this approach because the nature of our experiments required us to use mixed models, for which SS are not readily available. For partial Eta^2, output values need not necessarily sum to 1, as explained in the vignette for the analysis:
https://rdrr.io/cran/effectsize/f/vignettes/from_test_statistics.Rmd
We have added text at lines 372-373 to make clear that values needn't sum to 1, and that partial Eta^2 is an estimate from test statistics.

R^2
We thank the referee for this helpful suggestion regarding the coefficient of determination (R^2). Unfortunately we have not been able to enact it because:
- R^2 cannot be calculated for Cox models, which we used for development indices.
- For the linear models (fecundity, progeny, fertility) we have not been able to find a method to calculate R^2 that performs satisfactorially for all the different model classes we used: methods either generate errors, unrealistically high R^2 values (including R^2=1, which is not possible), or do not run on the class of model we use.
However, given the complexity of some of our mixed models, for which sums-of-squares are hard to identify/interpret, and therefore the way in which our effect size estimates were calculated (see above), we are not convinced that R^2 values would be informative in the way suggested (i.e. total variance explained / variance explained per factor).

Also with respect to development, how is the "index" calculated and why was development time not used as the measure? The methods indicate that every individual in a vial is being treated as an individual data point for development index? This is fine, but to avoid pseudo replication, a random vial effect needs to be included in the model to account for non-independence of individuals developing within the same culture vial.

We thank the referee for pointing out this important omission!

Development indices are simply the EMMs of a Cox model. We used this terminology because we believe it will be more intuitive for readers who are not versed in Cox models. Cox models integrate both time to event and nature of event (in this case, egg emerged to adult or not) into a single response variable. The EMM/development index is therefore an aggregate measure of both pieces of information, which simplifies interpretation and provides a statistical/visualisation framework that we can apply to both development and the other traits. We have added text at lines 268-272 to explain.

The development models include egg density per vial as a fixed effect. Since every vial has a unique egg count, additionally including vial is redundant and (according to our initial experiments with this approach) lead to an extremely poor fit. Egg count substitutes for vial number, and also accounts for rearing density. We have added this information into the materials and methods (lines 731-734)

The data seem not normally distributed with one DMN combination in each of the two feeding paradigms to be strong outlier. To what extent are the results in Fig 3 robust to versus driven by these particular combinations? These data are not included in some statistical models, but in the main text it would be good to summarize what patterns in Fig 3 are robust to versus driven by inclusion of particular genotypes in the statistical models.

The data and their distribution remain unchanged from the first round of review. We respond to the statistical and interpretative elements of this comment independently:

STATISTICAL
We recognise that the data are not normally distributed and we have been careful to use models that assume appropriate distributions. Specifically, as detailed in materials and methods, we have used negative binomial models for count data, binomial models for proportion data, and cox models for survival data. Furthermore the cox models are nonparametric. These methods are appropriate for the data distributions.

INTERPRETIVE
In figure 2 we present mitonucleogenotype-specific responses to dietary variation, with statistical analysis. These mitonucleotype-specific patterns of response underlie the overall mito-nuclear-diet effects and mitonucleogenotype-diet effects presented in figure 3; by showing specifically which populations do/do not respond to diet. Although we agree that it would be interesting to permute the data, including/excluding all possible combinations of genotypes, and measuring impacts on effect size, interpretation would not be practical given the complexity of our datasets and the number of different models we would need to fit (with 12 populations x 3 diets = 36 conditions, we calculate (2^36) combinations of experimental conditions, x4 traits x 2 feeding paradigms, giving approx. 550bn models and effect size calculations).

Does it not cause issues with the statistical models to have redundant information within mtDNA, nuclear genome, and the random factor of genotype?

This model remains unchanged from the first round of review.

The model is not problematic, because the random factor splits each combination of mitochondria * nucleus into independent populations. These are different pieces of information - the population/genotype variable specifies replicate populations, while mitochondria and nucleus show the properties of those populations. The different levels of the random factor are nested within each combination of mito * nuclear main effect. Thus, they are not redundant.

Furthermore, the mitonucleotype analysis is based purely upon the underlying sequence information, doing away with both the population/genotype random factor and the mito*nuclear interaction, but nevertheless leading to the same answer. This consistency across approaches to modelling mtDNA, nuclear genome and population/genotype, makes us confident of our results.

2) The last line in the abstract refers to the mt variant as a synonymous variant. It is also presented in figure 1 as a synonymous/non-coding variant. In the response to review, the authors acknowledge that the change in the rRNA is likely functional. Yet, this remains described as a synonymous or non-coding change in the abstract and at several place in the revised manuscript, including important arguments in the discussion. The synonymous/non-synonymous categorization is used to describe nucleotide changes in protein-coding genes. Non-coding is generally used to describe parts of the genome that do not encode genes (e.g., intergenic and potentially regulatory). tRNA and rRNA genes encode functional molecules, and, unlike in a protein-coding gene, we cannot assume that any variants are synonymous. Can the authors just call it a SNP in the lrRNA when they describe it and in figure 1, change the black highlighting to be nonsynonymous/RNA-encoding? I suggest

updating the last line of the introduction. Also, on Line 176, this should be non-protein coding. For line 394 of discussion ... I think this non-coding variation is different than SNPs in ribosomal RNA genes; the non-coding in this sentence is referring to the observation that there is much regulatory variation such as in intergenic regions of the genome that may have small RNAs or binding sites for regulatory factors or influence chromatin structure. Maybe a strict definition on "coding" means parts of the genome that use the genetic code to make proteins, but RNA genes are genic and encode functional RNA products that are distinct from the inter-genic, non-coding and presumably regulatory parts of the genome. Lines 449-453 of discussion should be reconsidered in light of this.

We have:
- updated the last line of the abstract
- added a new colouration to Fig 1G to specify when a SNP is in an RNA-encoding gene
- updated text throughout to specify non protein-coding

3) Lines 56-61; I would remove question "A." The authors can highlight this result from this study in the results and discussion, but the prior studies cited in line 55 use designs that address this question.

This question remains fundamental to our study, in order to set up our comparison of the effect size of DMN versus other terms, which as the referee notes, should be highlighted. We have therefore changed the way that these questions are introduced at line 54-55, removing comparison to prior studies.

4) While I understand what the authors intend by using nucleotype (to contrast with mitotype), I suggest using the term nuclear genome. My understanding of the term nucleotype is that it is used to describe ploidy variation in the nucleus (https://doi.org/10.1086/700636; https://pubmed.ncbi.nlm.nih.gov/6360135/). Mitonucleotypes could then be referred to as mitonuclear genotypes or genomes, which would be more consistent with the literature.

We appreciate the reviewer's point and care to ensure clarity. We have changed our terminology throughout, to the more specific terms mitogenotype, nucleogenotype, and mitonucleogenotype, defining them in the introduction at lines 62-65. We need to retain a specific term to reflect the concatenated form of the mitochondrial sequence and nuclear genome background that underpins a major and novel part of our analysis (e.g. Fig 3A). This terminology enables us to specify what different analyses do, while avoiding confusion with other terms in the literature.

5) In the original manuscript's presentation of the experimental design, it was not clear that the lines were genetically diverse, so I apologize that this question comes upon review of the revised manuscript. Why were genetically diverse lines (although presumably these were still from lab culture, so not as diverse as intentionally outbred populations) used? Or is the genetic variation just the residual heterozygosity that we expect in lab cultures of flies? Lines 95-96 state that "the crossing scheme was designed to produce distinct mitochondrial backgrounds bearing equivalent pools of standing nuclear variation." What is meant by "equivalent pools of standing nuclear variation?" Do you have data indicating that the A, B, and C nuclear genotypes have the same heterozygosity? What is the motivation for incorporating this genetic variation?

We used heterozygous populations because (A) that is the natural state of wild animals, and (B) the populations were the end result of a real-time evolution experiment, for which nuclear variation is required for selection to act upon. A substantial in-depth analysis of nDNA variation in that evolution experiment is in prep (Voigt et al), but beyond the scope of the present study, and beyond the scope of the short report format specified by the editors.

In figure 2 there are many genotype-diet combinations that are producing values of zero (and I think without error bars or very little variance). If the mito-nuclear genotypes are segregating in the introgressed populations and these genotypes are associated with the phenotype, shouldn't you also sample individuals with combinations of genotypes that are non-sterile or non-lethal? I understand that the mtDNA variants are primarily, although not completely, fixed differences between populations, but if there is backcrossing to the outbred nuclear background, shouldn't you expect interacting nuclear factors to be segregating in the pool of individuals that you phenotype? Otherwise, you would need to posit that nuclear factors associated with DMN effects are fixed differences between populations? At a minimum, this should be clearly explained for readers.

We address the statistical and interpretive points independently:

STATISTICAL
In the previous draft, all data points had confidence intervals, but sometimes the errors were very small and eclipsed by the point itself. The confidence intervals on development index data were particularly variable among conditions, because of the lethality of the diet to mitonucleogenotype 4.

For the avoidance of any doubt we have replotted this figure, now including the raw datapoints and boxplots to show distributions for the fecundity/progeny/fertility data. In response to both this point and the preceding comment about nature of the development index we have removed confidence intervals from that row of the plot, and because this better reflects the absolute nature of 100% mortality in mitonucleogenotype 4 (i.e. there is no error), while for points with quantitative information (i.e. all others) the CIs were invisibly small (as highlighted by the referee). We thank the referee for leading us to this solution. We believe this plot also makes clear the special nature of the development index and survival modelling. Materials and methods are edited at line 771 to clarify.

This new plot required additional space on the page to make the points clear, so we have moved the PCA to supplementary materials (now Figure S7).

INTERPRETIVE
Without a complete analysis of variation in the nuclear genomes, we make no assumptions about within-population variation. To us it seems possible that a treatment can be lethal to all individuals in a heterozygous population, yet not to a discrete heterozygous population, because segregating variation does not necessarily mean segregating variation in loci that confer stress resistance. In other words, populations can bear genetic variation, while remaining homozygous for loci that cause stress susceptibility/resistance. With respect to heterozygosity, the referee's comments in the first round of review were extremely helpful, making clear that in our revision we needed to carefully refer to our flies as grouped by population, but without making any claims about the underlying nDNA heterozygosity. We revised the manuscript as such, and our analyses were designed simply to accommodate nDNA as a factor, as described in materials and methods.

6) A characterized "Dahomey" mtDNA has diet-dependent effects on fitness in D. melanogaster (DOI: 10.1371/journal.pgen.1007735) and a male-sterile Dahomey mtDNA has been well characterized by co-authors on this manuscript. What is the relationship of the Benin mtDNA major alleles in this study and these characterized Dahomey mtDNA variants?

The Val161L mutation from Aw et al 2019 (DOI: 10.1371/journal.pgen.1007735) does indeed crop up in our flies also, we have edited text at lines 181-190 to elaborate. We thank the referee for pointing out this important point of reference.

We infer that the other variant the referee is the male sterility mutation at *Cyt-b*, reported in https://doi.org/10.1038/hdy.2011.12, https://doi.org/10.1111/eva.12297. However we detect some crossed wires here, as this mutation is found on a mitogenotype from Brownsville, Texas, which is not included in our experiment; and the mutation does not occur in the populations in our study. In those previous studies the mutation was most deleterious over the Benin nuclear background, which may underlie the mixup.

7) Line 207 indicates that there were eight distinct mitonuclear genome combinations which are labeled in Figure 1G. The mtDNA for mitonuc combination 4 is distinct at a number of positions from both other "orange" and other "purple" labeled mtDNA. It is also only present in the "orange" nuclear background.

This is effectively the same comment as comment #12, so we address it there.

Divergent diet-dependent phenotypes between groups 2 and 3 will show that mtDNA-diet effects are conditional on the nuclear background. Divergent diet-dependent phenotypes between groups 7 and 8 will also show that mtDNA-diet effects are conditional on the nuclear background.

We agree that these comparisons would indeed show these phenomena. Our plot makes these comparisons clear for those who are interested to make them, and indeed the referee has been able to do so on the basis of our existing plot.

However we feel that writing this level of granular analysis of specific comparisons would make for a very long read, which would not fit in the context of a short article. We also feel that these comparisons are not novel in the context of the literature, as the referee points out in other comments.

We are explicitly interested in diet-mito-nuclear interactions, and we show these both by modelling the interaction of diet with geographic origin of the mitogenotypes and the nucleogenotypes (i.e. DMN); and the interaction of diet with concatenation of the mtDNA sequence and nucleogenotype (i.e. diet:mitonucleogenotype). Specific comparisons between specific populations are not required to make that point. Our statistical models are carefully constructed to achieve this purpose - we find DMN and diet:mitonucleogenotype interactions across the full panel of populations, without recourse to specific pairwise comparisons.

Please see point above about impacts of specific populations and the astronomical number of tests that would be required to test this systematically.

But, it seems that the SNP that is highlighted in the manuscript is being analyzed for effects using contrasts between groups 5,6,7 and 8.

This is correct. We chose to focus specifically on this comparison because it allowed us to investigate some novel biology, and the fully-factorial design enabled by these specific populations supported an elegant fully-factorial design, nested within the bigger panel of populations.

This took me quite a while to piece together going back and forth between figures 1 and 2. It would be helpful for readers to clearly present what contrasts are being used for what inference in the results section. For example, mito-nuclear genotypes 1 and 4 are being used for what inference?

They are included in the mitonucleotype model and the geographic model. They are not involved in any further specific comparisons.

And is a DMN interaction specifically inferred as a diet-dependent genetic interaction using the subset of genotypes 5-8?

No, the DMN interaction in this subset of populations is used to infer a role for mitoribosomal 16s rRNA. Among these lines, the DMN interaction indicates a role for lrRNA, since no other mitochondrial SNPs are significantly different among these lines, as indicated in-text at lines 74-76, 224-239, 340-357.

I think this is the best way to define a DMN in this experimental design. Could patterns that support DMN effects be highlighted in figure 2 somehow?

The lettering at the top of the figure makes clear which populations are grouped into which mitonucleotypes. In our original submission we had the heatmap in Fig 1G alongside the data, making clear when the analysis was focussing on the geographic origin versus the sequence-based mitonucleotypes. We initially presented the geographic analysis and mitonucleotype analysis separately, but in the previous revision we rearranged into the current streamlined presentation following the referee’s previous request. We agree with the referee's view in the last round of comments, that this is the best presentation.

I found throughout the results section that DMN was used broadly and it was difficult for me to follow exactly what genetic-by-diet effects were being used to infer DMN effects. For example, both "diet:mitonucleotype" and "diet:mt:nuc" seem to be used equivalently as evidence of "DMNs"

diet:mitoNucleotype and diet:mito:nucleotype refer to different analyses, addressing different problems. We believe we make this clear in the manuscript. This comment implies that a more linear stepwise presentation of each analysis would be preferable. This was the original sequence of analyses in our manuscript which, following the referee's suggestion in the first round of review, we have now streamlined.

but I think only the latter is formally testing for diet by mtDNA by nuclear effects; if otherwise, then it should be clarified.
We disagree.

The latter analysis tests for interactions of the geographic origin of mitochondrial and nuclear genotypes with diet, in a fully-factorial design. It does not however tell us anything about the actual underlying sequence.

The former analysis goes a step further and reveals how specific differences in mitochondrial sequence modulate how nucleogenotypes respond to diet, but testing necessarily has to be per mitonucleogenotype because we do not have fully-factorial combinations of mitochondrial sequences and nucleogenotypes.

We would be happy to accept editorial guidance on any ways we could possibly make this more explicit.

8) Figure 2. Are the p-values reported in Fig 2 multiple-test corrected?

Yes, they are Tukey corrected. We have specified in the legend at line 1231.

9) Lines 241-247; also to clarify another reviewer's question, are parents developed on the different diets or only exposed as adults to the different diets before they lay eggs on the experimental and then standardized diets? Clarity on this allows the reader to infer if the effect of diet on parents and their progeny is potentially via effects on the development of parent gonadal tissue and/or via effects of diet on gametogenesis in parents. Is the effect of chronic versus parental diet confounded with the parental age at which an offspring was laid? I am willing to believe that effects are likely dietary, but good to mention this as a caveat.

Yes, all flies developed on the same diet before being switched to experimental diet as adults, prior to phenotyping resulting eggs and offspring. We have added text at lines 248-251 to clarify.

10) In the results section, include statistical evidence (p-value, preferably multiple-test corrected) for statements such as in line 259 "mitonucleotype determined magnitude," and for the diet-by-mitonucleotype interaction in the GLM. Similar in line 262. Line 328-329 is where it would be important to report p-values for the mt-by-nuclear-by-diet interaction using the subset of genotypes 5-8.

At all points when statistical analyses have been conducted, we refer the reader to supplementary tables, containing all statistical analyses. Our view is that reporting eight separate sets of F-values, DF and P-values (i.e. one per trait) would disrupt the manuscript's flow, and add little beyond our existing presentation. However we are again happy to accept editorial advice on journal style.

The statement at 259 (now 282-283) in full is "Chronic lipid feeding was deleterious for all traits, but mitonucleotype determined magnitude". To provide direct statistical evidence of this we would need to fit an entirely new set of models, excluding EAAs, and then of course do the same excluding lipid to enable equivalent comparison of the effects of EAAs. This would treble the number of analyses. We do not feel that the insight this would add justifies the additional complexity. In the case of this specific comparison, it is visually clear that the differences between control and +lipid conditions vary substantially by mitonucleotype. However we are happy again to accept editorial advice.

11) Multiple places in the manuscript report that the C/T SNP is "sufficient to cause" DMN variation. The SNP seems "associated with" or "involved in" DMN interactions. With an epistatic mutation, one SNP alone is not sufficient; it seems by definition to also require variation in the nuclear genome and in dietary environment. This is more of an association study so the language of association seems more appropriate.

We have altered the text to add the word "associating" (see line 419). However it is important to also retain the original terminology: we maintain that "sufficient" is the correct term since this SNP was the only difference between the specific mitochondria in question. It alone can alter phenotype, without other significantly differentiated SNPs. We have edited the text to make clear that we are referring specifically to the sufficiency to drive epistasis. Sufficiency and necessity are common terminology in genetics which are appropriate to our subject matter.

12) A final point is that I really don't know what to make of that AA3 mtDNA. Are the authors not perplexed by how this mtDNA from a putatively Australian lineage/culture in the lab appears recombinant with the Benin mtDNA sequence?

We have added text at lines 128-133 to ensure that we acknowledge AA3's mitochondria, and discuss the possible origin of differences from AA1-2. We took an agnostic starting position and we report what we found precisely. We cannot reject sequencing results because of prior expectation, our analyses are robust, and geographic origin is anyway superceded when we base our analysis on sequence.

In the geographic analysis, is it considered an Australian mtDNA even though the tree in Fig 2G groups it with the Benin mtDNA?

Yes. It is designated Australian because of its geographic origin in Australia. The sequencing results then specify SNPs exactly, and their frequencies. We analyse it both ways - this is a strength of our analysis and a new standard in the field.

Are the statistical results and conclusions robust to leaving this mtDNA out of the geographic analyses of if you consider this to be a Benin allele? I am really curious because it differs from Australia at many sites, and in each case it has the Benin allelic state, which is not what I would expect.

We have dealt with the problem already, through our sequence-based mitonucleogenotype analysis. By analysing per sequence, the differences between AA3 and AA1-2 are accounted for, and their geographic origins become irrelevant. In other words there is nothing to account for. An analogy would be in applying human genomics to disease - when looking for disease-associated SNPs, we do not say that patients have African or Australian genomes, and then base treatment on that: we simply look for the SNP. Taking this sequence-informed approach is a new standard in the field, which - as requested - we have streamlined and emphasised, and backtracking to using geographic origin as a proxy for variation would negate the progress we have made by using sequence.

Minor comments:

Lines 585-587; this description of the diet manipulation was unclear.

We have revised the text (now at lines 617-619) to make more concise and clear.

Methods; I have not done a ChIP-seq experiment, so was unfamiliar how sequence data from a ChIP-seq experiment can be used for estimating allele frequencies as in a pooled-sequencing design. Readers may benefit from more details on this and whether the resulting coverage is within ranges that are considered good for estimating allele frequencies.

ChIP-seq input controls are basically whole-genome DNA extractions, fragmented for sequencing. DNA was extracted from pools of 50 individual flies, which is above the recommended minimum number of pooled individuals in a Pool-seq experiment and sequencing depths were also sufficient for a Pool-seq setup, both similar to those in the recent DrosEU Pool-seq paper (Kapun et al. 2020 - https://doi.org/10.1093/molbev/msaa120), which includes sequencing depths. Sequencing was done paired-end with a read length of 75bp, also as recommended for Pool-seq.

We have ensured that all relevant details and variables in our analysis are included in materials and methods. We have also referenced the relevant literature, which direct the reader toward standards in this area. Our manuscript focusses on the biology that we have revealed using these established methods. It would be beyond its scope to discuss the methods.
