# Supplementary material for: Mitonuclear interactions shape both direct and parental effects of diet on fitness and involve a SNP in mitoribosomal 16s rRNA
Source: PLoS Biol. 2023 Aug 21;21(8):e3002218. doi: 10.1371/journal.pbio.3002218 (PMC10441796; doi:10.1371/journal.pbio.3002218)
Supplement: Attachment — Submitted filename: 127022_1_rebuttal_2389737_rdsq53.docx [file pbio.3002218.s036.docx]

**Manuscript number:** #RC-2022-01476

**Corresponding author(s):** Adam Dobson

*[Please use this template only if the submitted manuscript should be considered by the affiliate journal as a full revision in response to the points raised by the reviewers.*

*If you wish to submit a preliminary revision with a revision plan, please use our "*[*Revision Plan*](https://docs.google.com/document/u/0/d/1HHp62Tfeb44nd3Gu_3ChB0b8gmMdEog7GjZcZFwHSiQ/edit)*" template.* ***It is important to use the appropriate template to clearly inform the editors of your intentions.****]*

## 1. General Statements [optional]

We thank the referees for taking the time to review our manuscript thoroughly. We are glad to see that the important points of our study came across clearly, and that they did not feel that significant revisions are required to support our findings.

## 2. Point-by-point description of the revisions

**Reviewer #1**

Page 3, line 35 - you state that variation in mitochondrial function can contribute to variation in dietary optima. I would add a citation here.

We have added an exemplar citation.

Page 4, line 74 - If possible, I would add a little more clarifying detail regarding the crossing scheme to the main text. I would just be clear that the generation of these mitonucleotypes takes advantage of the maternal transmission of the mitochondrial genome by crossing virgin females from population 1 to males from population 2, followed by continual backcrossing of virgin females each generation to males from population 2. You go into specific details in the materials & methods section, but I was surprised not to see it earlier. I know this is probably more detail, but I think it's better to be very clear about the crossing scheme used.

We have added this information at lines 72-75.

Page 5, line 90 - I would move the citations for the fact that enriching essential amino acids promotes fecundity from the supplement to the main text. You say that this is an established manipulation, and it would be best to point to examples of this manipulation, especially as some of your results find that fertility is negatively affected.

Citations have been added.

Page 8, line 129 - In this paragraph, you speak about the impacts of chronic EAA feeding in the AA, BA, and AB mitonucleotypes, but you do not coment on BB? Is there a reason for this? (This connects to one of my comments later)

We thank the referee for pointing this out, and we have edited the text (lines 137-139) to include the *BB* populations.

General Questions - This may be outside the scope of your study, but I was surprised that there was no commentary on co-adaptation (or the potential lack of) between the mitochondrial and nuclear genome in the discussion. It's not immediately clear to me how isolated and for how long the Australian and Benin populations are, but I would expect there to be co-evolution/co-adaptation between the mitochondrial and nuclear genomes. Consequently, I would have expected AA and BB populations to show elevated fitness values; however, it actually seems as if though the AA mitonucleotype performs the worst when given the chronic EAA diet. I wonder if you could comment on the co-evolutionary potential between these two genomes.

We have added a new paragraph to the discussion at lines 289-297, to address this point.

**Reviewer #2**

- Have the authors thought about looking at rates of food consumption, and could some populations be consuming less (or none) of the experimental diets, possibly explaining lethality?

We thank the referee for pointing out this potential mechanism. We have not measured feeding in our mitolines, as we feel that detailed mechanistic characterization is a subject for future work and beyond the scope of the present study. We have instead added this putative mechanism to the discussion at lines 342-344.

- Line 136: this study's data does indeed show diet-dependent effects of mitonuclear interactions, but it not the first one, maybe rephrase? See refs (a couple are cited in the supplementary text, but it would be useful to add to the main text):

Aw, W. C. et al (2018). PLoS Genetics doi:10.1371/journal.pgen.1007735

Camus, M. F., Moore, J., & Reuter, M. (2020). Biology Letters, 16(2), 20190891. doi:10.1098/rsbl.2019.0891

Camus, M. F., Kotiadis, V., Carter, H., Rodriguez, E., & Lane, N. (2022). bioRxiv, doi:10.1101/2022.02.10.479862

Cormier, R. P. J. et al. (2019). Scientific Reports. doi:10.1038/s41598-018-36060-5

Rodríguez, E. et al. (2021). Frontiers in Genetics. doi:10.3389/fgene.2021.734255

Towarnicki, S. G., & Ballard, J. W. O. (2018). Frontiers in Genetics. doi:10.3389/fgene.2018.00593

We think this comment may come from a lack of clarity around what we were trying to express, and so we have modified the text. We agree that a number of prior publications have shown DMN effects, and we have updated citations as suggested. However these prior studies have focussed on physiology and evolution, and we are not aware of prior studies that have shown that mitonuclear incompatibility is diet-dependent. We hope that the additional text at lines 141-144 make this distinction clear.

- Figure 1D and results section lines 156-178: why is the term "eggs" included in development? I assume it refers to the measure of the amount of eggs, could the authors explain and mention it in the main text and/or figure legend? And why is it "eggs+1" in fig S2D, I could not find an explanation in the methods for this difference in the label.

In light of this comment we can see how our previous phrasing may have been confusing, and we have updated accordingly. To assay development time, we allowed flies to lay eggs, counted the eggs, then allowed the eggs to develop. Since the dietary manipulations modulated egg laying, and rearing density might impact development indices, we wanted to include a measure of density as a covariate in our development analyses. We originally phrased this literally as “eggs”, we have now replaced it with “rearing density”, which we hope is more context-appropriate and less confusing.

Regarding the comment on “+1” in axis labels: for count data (eggs and progeny), in some cases we present the data on a log scale. It is necessary to add one in these cases so that the log data can be plotted (i.e. to avoid attempting to log zero). We have added text to figure legends to make clear when the data are plotted as x+1.

- Line 221, last sentence of the paragraph: I would make it clearer that this was in the "chronic" paradigm, I found it difficult to follow here.

“Chronic” added to clarify (line 228)

- Line 446 typo in the figure legend, should read "24h on..."

“Son” corrected to “on”.

- Figure 2A: "Genome origin" section of the legend could be placed on the top of the legend, I would find it easier to see as we read the figure from left to right.

Legend moved as suggested.

- Figure 2B: some kind of separation between T and C (dashed lines for eg.) would be appreciated to be able to compare better at a glance.

We have added dashed lines and updated the legend to reflect the update.

- Figure S2D: boxplot lines seem very thin, could the outline be made clearer? It was especially difficult to see on a printed version.

We thank the reviewer for pointing this out. The boxes are now darker.

- Line 506, S2B legend: should "Benin" be used instead of "Dahomey"? Keeping the Dahomey mentioned only in Supporting text line 153.

We thank the reviewer for pointing this out and have updated the supplementary text accordingly.

Figure S3B and its legend text: the diets now appear completely different, except for EAA. Why is it "sya", should it be the same as "rearing diet" in S2B, or is it the "control" diet? "Mar", margarine, is to my understanding the lipid diet mentioned throughout the paper? I assume this is a matter of fixing the legend labels, unless I misunderstood this part. In the legend text lines 532-536, I could not find any explanation for this difference in labels.

We thank the reviewer for highlighting this error which is indeed a simple difference between nomenclature used in R code and in the manuscript. We have remedied the labels to match the manuscript.

- Supplementary text line 43: "wild-type" meaning Benin flies?

Yes. We have updated the supplementary text at lines 39-51 and lines 181-186 to clarify the provenance of these flies and how diets were fed.

- Supp. text line 46: "development" medium is sya? See comment above about confusing diet names.

We thank the referee for pointing out that our preceding names were confusing. We have harmonized names throughout the manuscript, and added additional text in materials and methods (supplementary text lines 134 – 141) to better explain. We have also added paragraph breaks with development medium and experimental media in materials and methods (supplementary text lines 142 and 146) to highlight the distinction.

- Supp. text line 51: I think the figures referenced are not the correct ones (S2B and S2C)? Should they be S2D and S2E?

Corrected.

- Supp. text line 64: "AA1" is repeated twice in the parentheses, should it read "AA3" instead?

Corrected.
